# Study on the Melting Temperature of CaF_2_-CaO-MgO-Al_2_O_3_-TiO_2_ Slag under the Condition of a Fixed Ratio of Titanium and Aluminum in the Steel during the Electroslag Remelting Process

**DOI:** 10.3390/ma14206047

**Published:** 2021-10-13

**Authors:** Dong Hou, Peng Pan, Deyong Wang, Shaoyan Hu, Huihua Wang, Ganggang Zhang

**Affiliations:** 1 School of Iron and Steel, Soochow University, Suzhou 215000, China; houdong0702@suda.edu.cn (D.H.); panpengxy@163.com (P.P.); dywang@suda.edu.cn (D.W.); 2 Digital Campus, Capital Normal University, Beijing 100000, China; zgg@cnu.edu.cn

**Keywords:** electroslag remelting, melting temperature of slag, steel containing titanium, thermodynamics, phase diagram

## Abstract

During the process of electroslag remelting (ESR) of steel containing titanium and aluminum, the activity ratio between titania and alumina in CaF_2_-CaO-MgO-Al_2_O_3_-TiO_2_ slag must be fixed in order to guarantee the titanium and aluminum contents in the ESR ingots. Under the condition of fixed activity ratio between titania and alumina in the slag, the melting temperature of slag should be investigated to improve the surface quality of ESR ingots. Therefore, this paper focuses on finding a kind of slag with low melting temperature that can be used for producing steel containing titanium. In the current study, the thermodynamic equilibrium of 3[Ti] + 2(Al_2_O_3_) = 4[Al] + 3(TiO_2_) between SUS321 steel and the two slag systems (CaF_2_:MgO:CaO:Al_2_O_3_:TiO_2_ = 46:4:25:(25 − *x*):*x* and CaF_2_:MgO:CaO:Al_2_O_3_:TiO_2_ = 46:4:(25 − 0.5 *x*):(25 − 0.5 *x*):*x*) are studied in an electrical resistance furnace based on Factsage software. After obtaining the equilibrium slag with fixed activity ratio between titania and alumina, the melting temperatures of the two slag systems are studied using slag melting experimental measurements and phase diagrams. The results show that the slag systems CaF_2_:MgO:CaO:Al_2_O_3_:TiO_2_ = 46:4:25:(25 − *x*):*x*, which consists of pre-melted slag S0 (CaF_2_:MgO:CaO:Al_2_O_3_ = 46:4:25:25) and pre-melted slag F1 (CaF_2_:MgO:CaO:TiO_2_ = 46:4:25:25), can not only control the aluminum and titanium contents in steel, but also have the desired low melting temperature property.

## 1. Introduction

Electroslag remelting (ESR) [1,2] is one of the processes used to produce high quality special steels. During ESR process, the slag plays important roles in chemical composition and surface quality of ingot. On the one hand, the slag CaF_2_-CaO-MgO-Al_2_O_3_-TiO_2_ should have the fixed activity ratio of $\mathrm{lg}\left(a_{{\mathrm{TiO}}_{2}}^{3}/a_{{\mathrm{Al}}_{2}O_{3}}^{2}\right)$ to guarantee the thermodynamic equilibrium of 3[Ti] + 2(Al_2_O_3_) = 4[Al] + 3(TiO_2_) and the ratio of $\mathrm{lg}\left(w_{\left[\mathrm{Ti}\right]}^{3}/w_{\left[\mathrm{Al}\right]}^{4}\right)$ in steel. On the other hand, the slag should also have a low melting temperature to improve the surface quality of the ESR ingots. Especially for superalloy or stainless steel with a melting temperature lower than 1370 °C (1643 K), the melting point of the slag used for ESR of superalloy and stainless steel should be lower than 1270 °C (1543 K). Therefore, it is essential to investigate the melting temperature of CaF_2_-CaO-MgO-Al_2_O_3_-TiO_2_ slag under the condition of fixing activity ratio of titania and alumina in the slag during the ESR process.

The studies on CaF_2_-CaO-MgO-Al_2_O_3_-TiO_2_ slag used for steel containing titanium are mainly divided into two categories: one is about the effect of TiO_2_ on the physical property of slag, and the other is about the effect of each slag component on the activities of TiO_2_ and Al_2_O_3_. Shi [3,4,5] studied the effect of TiO_2_ on the crystallization behavior of CaF_2_-CaO-Al_2_O_3_-MgO-TiO_2_ slag and pointed out that TiO_2_ has large effect on the physical property of the slag. Duan [6,7] studied the effect of each slag component on the activities of Al_2_O_2_ and TiO_2_, and determined an appropriate slag to be used for ESR of superalloys based on experiments and thermodynamics. Jiang [8,9,10,11,12,13,14,15,16] investigated the thermodynamic equilibrium of 3[Ti] + 2(Al_2_O_3_) = 4[Al] + 3(TiO_2_) and the effect of slag components on activities of Al_2_O_2_ and TiO_2_ in CaF_2_-CaO-MgO-Al_2_O_3_-TiO_2_ slag system, and then TiO_2_ in slag was calculated to control the titanium and aluminum contents in ESR ingot. However, the above researches [17,18,19,20,21] did not comprehensively consider the physical properties and thermodynamic equilibrium of 3[Ti] + 2(Al_2_O_3_) = 4[Al] + 3(TiO_2_). Under the condition of controlling the titanium and aluminum contents in steel, the optimized slag with low melting temperature cannot be acquired according to the studies above.

To the best of the authors’ knowledge, under the condition of fixing activity ratio between titania and alumina in slag, investigation on the melting temperature of slag has not been reported so far. In the present work, the thermodynamic equilibrium of 3[Ti] + 2(Al_2_O_3_) = 4[Al] + 3(TiO_2_) was studied by the slag-metal reaction in a resistence furnace and the Factsage software. After obtaining the equilibrium slag with fixed activity ratio between titania and alumina, the melting temperature of CaF_2_-CaO-Al_2_O_3_-MgO-TiO_2_ slag was studied by slag melting experimental measurements and phase diagram. At last, the slag design diagram consisting of $\mathrm{lg}\left(a_{{\mathrm{TiO}}_{2}}^{3}/a_{{\mathrm{Al}}_{2}O_{3}}^{2}\right)$ isoactivity lines and slag phase diagram (CaF_2_:MgO:CaO:Al_2_O_3_:TiO_2_ = 46:4:*x*:*y*:*z*, *x* + *y* + *z* = 50) was made for acquiring the optimized CaF_2_-CaO-Al_2_O_3_-MgO-TiO_2_ slag with low melting temperature.

## 2. Experimental

### 2.1. Slag-Metal Reaction Experiments in Resistance Furnace

**Figure 1:**
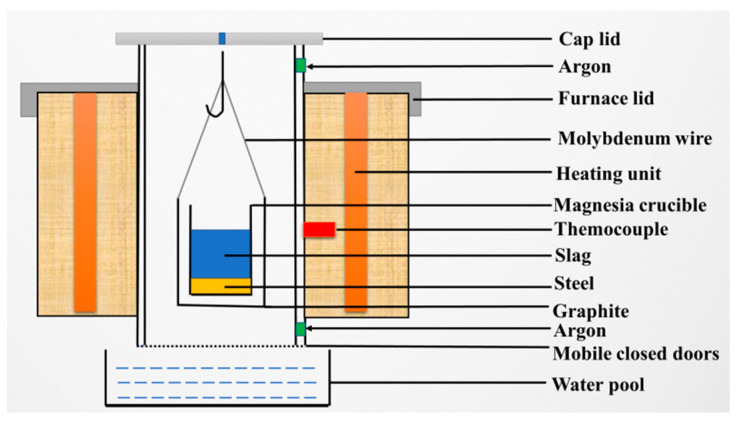
Schematic diagram of resistance furnace with function of dropping crucible from bottom.

SUS321 stainless steel produced by Dongbei special steel group Co. Ltd, Dalian, China was used in current study. Its chemical composition is listed in Table 1. The chemical compositions of Slag S0F1-82, S0F1-64, S0F2-82 and S0F2-64 are listed in Table 2, and the chemical compositions of pre-melted slag S0, F1 and F2 are listed in Table 3. Each slag-metal reaction experiment is carried out with 80 g slag and 50 g steel by using a resistance furnace, as shown in Figure 1. The heating unit is made of molybdenum disilicide. The temperature of the liquid metal is continuously measured by means of a B-type reference thermocouple produced by Kejing material technology Co. Ltd, Hefei, China. Argon is used to protect the slag-metal reaction system from top and bottom of the furnace at the rate of 2 Nl/min.

The experimental procedures can be described as follows. Firstly, 50 g of steel and 80 g of slag are placed into a MgO crucible with 30 mm in inner diameter and 70 mm in depth. Then the crucible is placed in a graphite crucible with molybdenum wire for suspension. After the whole crucible is placed in the chamber, the power is switched on and the furnace is heated to the experimental temperature (1823 K (1550 °C)) at a rate of 8 K/min.

After the furnace temperature was held for 60 min at 1823 K (1550 °C) [8,9], the crucible was dropped into liquid water quickly. The contents of Si, Al and Ti in each steel sample are analyzed by the inductively coupled plasma-mass spectroscopy (ICP-MS) technique and the concentrations of Al_2_O_3_, TiO_2_ and MgO in slag samples are analyzed by inductively coupled plasma-atomic emission spectroscopy (ICP-AES). The results are listed in Table 4.

### 2.2. Slag Melting Temperature Tests

During industrial ESR of steel containing different titanium and aluminum contents process, the TiO_2_ powder combined with pre-melted slag CaF_2_-CaO-Al_2_O_3_-MgO are added into the water cooling molds of the ESR furnace. In order to prevent the TiO_2_ powder from volatilizing with the air flow during the slag addition process, a new pre-melted slag S0 without TiO_2_ and a pre-melted slag F1(F2) with high TiO_2_ are designed. Their compositions are listed in Table 3. By mixing S0 and F1 in the ratio of 88:12, the slag S0F1-1 in Table 3 was acquired. Slag S0F1-2 and S0F1-3 can be acquired when the ratios of S0:F1 are 76:24 and 64:36, respectively. Slag S0F2-1, S0F2-2 and S0F2-3 can be acquired when the ratios of S0:F2 are 88:12, 76:24 and 64:36, respectively.

**Figure 2:**
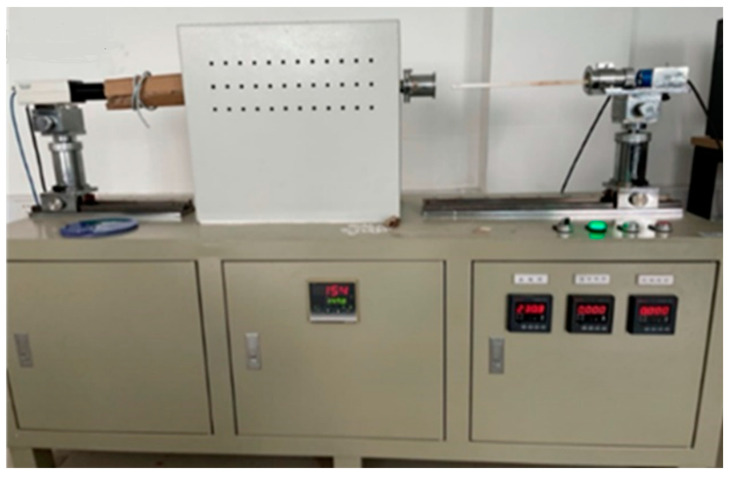
Test facility for determination of slag melting behavior.

Slag melting experiments were carried out by using a high temperature specimen deformation method. A diagram of the test system is shown in Figure 2. In order to evaluate the melting behavior, the pre-melted slag powders were compressed into cylindrical samples of 3 mm diameter and 3 mm high. For each test, the slag sample was placed at the centre of a corundum substrate which was then located within the hot zone of a molybdenum wire furnace. The furnace was heated at 10 °C/min up to the slag melting temperature, which is defined as the temperature at which the cylindrical specimen attained a hemispherical shape. The melting temperature of slag was measured using a high temperature microscope, and the results are listed in Table 3.

## 3. Results and Discussion

### 3.1. Slag-Metal Reaction Experiments Results

The results of slag-metal reaction experiments in resistance furnace are shown in Table 4. Due to the existence of unstable oxides SiO_2_ and FeO, both aluminum and titanium are lower than them in the steel before experiments. If assuming that the slag-metal reaction of 3[Ti] + 2(Al_2_O_3_) = 4[Al] + 3(TiO_2_) in Table 4 reaches thermodynamic equilibrium [8,9], the activity coefficients of alloy element in steel and oxide component in slag can be experimental measured based on thermodynamics. At the slag-metal interface under 1550 °C, the following Reaction (1) will take place [22,23]. After substituting Ti, Al, Al_2_O_3_ and TiO_2_ of Table 4 into Equation (2), the activity coefficient value of Equation (2) in Exp.S0F1-82, Exp.S0F2-82, Exp.S0F1-64 and Exp.S0F1-64 are experimental measured as −3.57, −3.21, −3.42 and −2.89, as shown in Table 4.

3[Ti] + 2(Al_2_O_3_) = 4[Al] + 3(TiO_2_) (1)

(2) $$\mathrm{lg}K=\mathrm{lg}\frac{a_{\mathrm{Al}}^{4}\cdot a_{{\mathrm{TiO}}_{2}}^{3}}{a_{\mathrm{Ti}}^{3}\cdot a_{{\mathrm{Al}}_{2}O_{3}}^{2}}=\mathrm{lg}\frac{w_{\left[\mathrm{Al}\right]}^{4}\cdot X_{{\mathrm{TiO}}_{2}}^{3}}{w_{\left[\mathrm{Ti}\right]}^{3}\cdot X_{{\mathrm{Al}}_{2}O_{3}}^{2}}+\mathrm{lg}\frac{f_{\mathrm{Al}}^{4}\cdot \gamma_{{\mathrm{TiO}}_{2}}^{3}}{f_{\mathrm{Ti}}^{3}\cdot \gamma_{{\mathrm{Al}}_{2}O_{3}}^{2}}=-\frac{35300}{T}+9.94$$

where $a_{{\mathrm{TiO}}_{2}}$ and $a_{{\mathrm{Al}}_{2}O_{3}}$ are the activities of TiO_2_ and Al_2_O_3_ in the slag; $X_{{\mathrm{TiO}}_{2}}$ and $X_{{\mathrm{Al}}_{2}O_{3}}$ are the mole fraction of TiO_2_ and Al_2_O_3_ in slag; $\gamma_{{\mathrm{TiO}}_{2}}$ and $\gamma_{{\mathrm{Al}}_{2}O_{3}}$ are the activity coefficients of TiO_2_ and Al_2_O_3_ in slag; *f*_Al_ and *f*_Ti_ are the activity coefficients of Al and Ti; $\mathrm{lg}\frac{f_{\mathrm{Al}}^{4}\cdot \gamma_{{\mathrm{TiO}}_{2}}^{3}}{f_{\mathrm{Ti}}^{3}\cdot \gamma_{{\mathrm{Al}}_{2}O_{3}}^{2}}$ is the activity coefficient of Equation (2).

**Figure 3:**
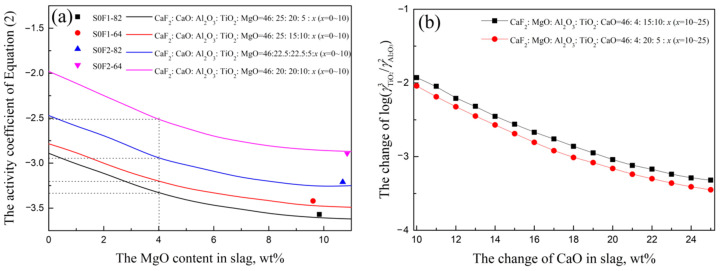
(**a**) The change of activity coefficient of Equation (2) with MgO, and (**b**) the change of lg($\gamma_{{\mathrm{TiO}}_{2}}^{3}/\gamma_{{\mathrm{Al}}_{2}O_{3}}^{2}$) with CaO in slag calculated by Factsage.

During the slag-metal reaction experiments, the MgO in slag after experiments was increased to 10% because of MgO crucible being eroded by slag. In order to investigate the thermodynamic equilibrium of SUS321 steel and slag S0-F1(F2) further, the activity coefficients of Ti and Al in steel are calculated by Equation (3) and the value of $\mathrm{lg}\left(f_{\left[\mathrm{Ti}\right]}^{3}/f_{\left[\mathrm{Al}\right]}^{4}\right)$ is considered as −0.12. The interaction parameters [24,25,26] used in present study are listed in Table 5. The activity coefficients of TiO_2_ and Al_2_O_2_ in slag are calculated based on Factsage 7.3-FToxid FactPS. The change of activity coefficient of Equation (2) with MgO in Exp.S0F1-82, Exp.S0F2-82, Exp.S0F1-64 and Exp.S0F1-64 are calculated, as shown in Figure 3a. It is clear that the calculated results in Figure 3a are in good agreement with measured results listed in Table 4.

(3) $$\mathrm{lg}f_{i}=\sum e_{i}^{j}w[\%j]$$

**Figure 4:**
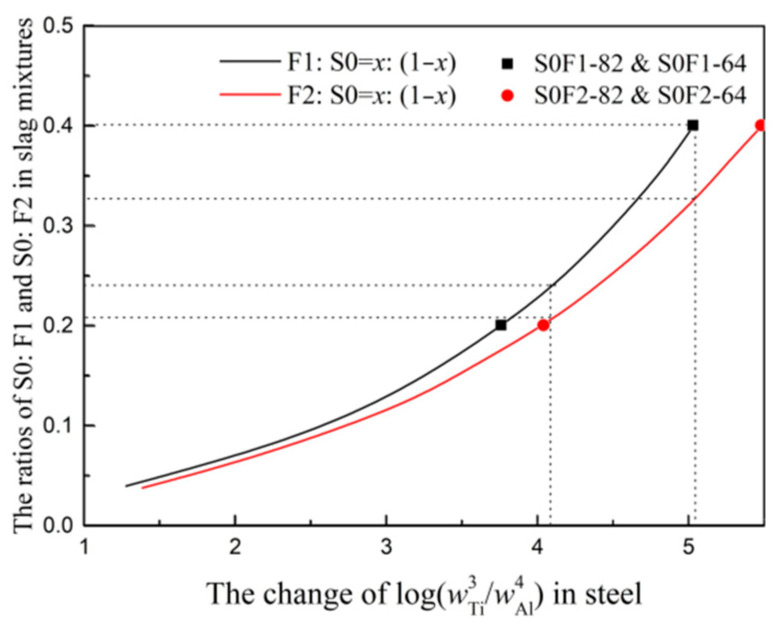
The changes of S0:F1 and S0:F2 ratios in slag mixtures with lg($w_{\left[\mathrm{Ti}\right]}^{3}/w_{\left[\mathrm{Al}\right]}^{4}$) in steel.

Figure 3a shows that the activity coefficient values of Equation (2) under slag S0:F1 = 8:2, S0:F1 = 6:4, S0:F2 = 8:2 and S0:F2 = 6:4 can be calculated as −3.33, −3.20, −2.95 and −2.52, as listed in Table 6. After obtained the activity coefficient value of Equation (2) in each slag, the slag S0:F1 = 8:2, S0:F1 = 6:4, S0:F2 = 8:2, S0:F2 = 6:4 and corresponding $\mathrm{lg}\left(w_{\left[\mathrm{Ti}\right]}^{3}/w_{\left[\mathrm{Al}\right]}^{4}\right)$ are calculated in Table 6, which will be used as points in Figure 4.

It is also can be seen that the activity coefficients of Equation (2) in each slag listed in Table 6 are different, it is different CaO content in slag that changes the activity coefficients of TiO_2_ and Al_2_O_3_, which has been studied based on ion and molecular coexistence theory (IMCT) in the previous study [8,9]. The changes of $\mathrm{lg}\left(\gamma_{{\mathrm{TiO}}_{2}}^{3}/\gamma_{{\mathrm{Al}}_{2}O_{3}}^{2}\right)$ with CaO are calculated based on Factsage software, as shown in Figure 3b. The $\mathrm{lg}\left(\gamma_{{\mathrm{TiO}}_{2}}^{3}/\gamma_{{\mathrm{Al}}_{2}O_{3}}^{2}\right)$ decreases with the increase of CaO in slag, which means the ratio of $\mathrm{lg}\left(w_{{\mathrm{TiO}}_{2}}^{3}/w_{{\mathrm{Al}}_{2}O_{3}}^{2}\right)$ in slag should increase with the increase of CaO under the condition of fixed $\mathrm{lg}\left(\gamma_{{\mathrm{TiO}}_{2}}^{3}/\gamma_{{\mathrm{Al}}_{2}O_{3}}^{2}\right)$ in slag.

The changes of S0:F1 and S0:F2 ratios in slag mixtures with $\mathrm{lg}\left(w_{\left[\mathrm{Ti}\right]}^{3}/w_{\left[\mathrm{Al}\right]}^{4}\right)$ are calculate according to Equations (1)–(3) and Factsage software at the temperature of 1550 °C, as shwon in Figure 4. It is can be seen that the points listed in Table 6 are in good agreement with the calculated results based on thermodynamics and Factage software. The slag S0-F1 containing high CaO needs large ratio of $\mathrm{lg}\left(w_{{\mathrm{TiO}}_{2}}^{3}/w_{{\mathrm{Al}}_{2}O_{3}}^{2}\right)$ to guarantee the thermodynamic equilibrium of 3[Ti] + 2(Al_2_O_3_) = 4[Al] + 3(TiO_2_) and the ratio of $\mathrm{lg}\left(w_{\left[\mathrm{Ti}\right]}^{3}/w_{\left[\mathrm{Al}\right]}^{4}\right)$ in steel.

If the titanium and aluminum contents in steel are given, the mixture ratios between pre-melted slag S0 and F1(F2) can be acquired according to Figure 4. In order to compare the melting temperature of two slag systems S0-F1 and S0-F2 under the condition of fixing the titanium and aluminum contents in steel, the steel with $\mathrm{lg}\left(w_{\left[\mathrm{Ti}\right]}^{3}/w_{\left[\mathrm{Al}\right]}^{4}\right)\;$4.10 and 5.03 combined with corresponding slag systems S0-F1 and S0-F2 are calculated, as shown in Table 7.

Then the melting temperatures of thermodynamic equlibrium slag systems in Table 7 are measured in slag melting experiments, and the results are listed in Table 3.

### 3.2. Slag Melting Temperature Results

**Figure 5:**
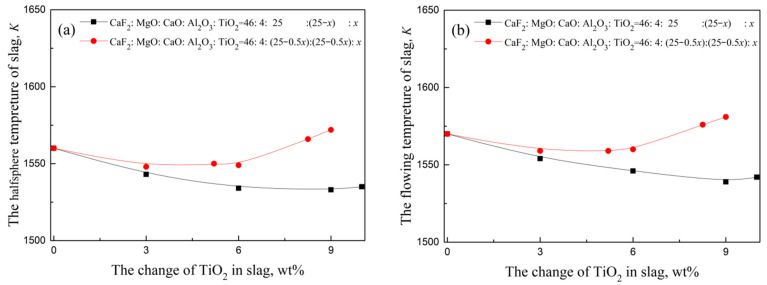
The change of melting temperature with different S0:F1 and S0:F2 ratios: (**a**) halfsphere temperature, and (**b**) flowing temperature.

The halfsphere melting temperature and flowing melting temperature results for the slag listed in Table 3 are shown in Figure 5. It is clear that: (i) with a CaO/(Al_2_O_3_ + TiO_2_) ratio = 1, the melting temperature of the slag S0-F1 is lower than slag S0-F2 with a CaO/Al_2_O_3_ ratio = 1 under the condition of increasing TiO_2_ content in slag; (ii) when the TiO_2_ content reaches more than 9%, the melting temperatures of slag S0F1-3 and S0F1-4 are much lower than that of slag S0F2-4 and S0F2-5; (iii) the melting temperatures of slag S0F1-4 is much lower than that of slag S0F2-4 in Table 7 under the condition of fixing steel with $\mathrm{lg}\left(w_{\left[\mathrm{Ti}\right]}^{3}/w_{\left[\mathrm{Al}\right]}^{4}\right)$5.03; (iv) the melting temperature of the slag S0-F2 system (CaO/Al_2_O_3_ ratio = 1) decreases first and then increases with the increase of TiO_2_ content, which has been described in detail based on SHTT, SEM and XRD in [3]. As the description of conclusion in [3], TiO_2_ addition from 0 to 6.43 mass% inhibited crystallisation behaviour of CaF_2_-CaO-MgO-Al_2_O_3_ ESR type slag, whereas the further TiO_2_ addition up to 9.73 mass% greatly enhanced the crystallisation tendency.

**Figure 6:**
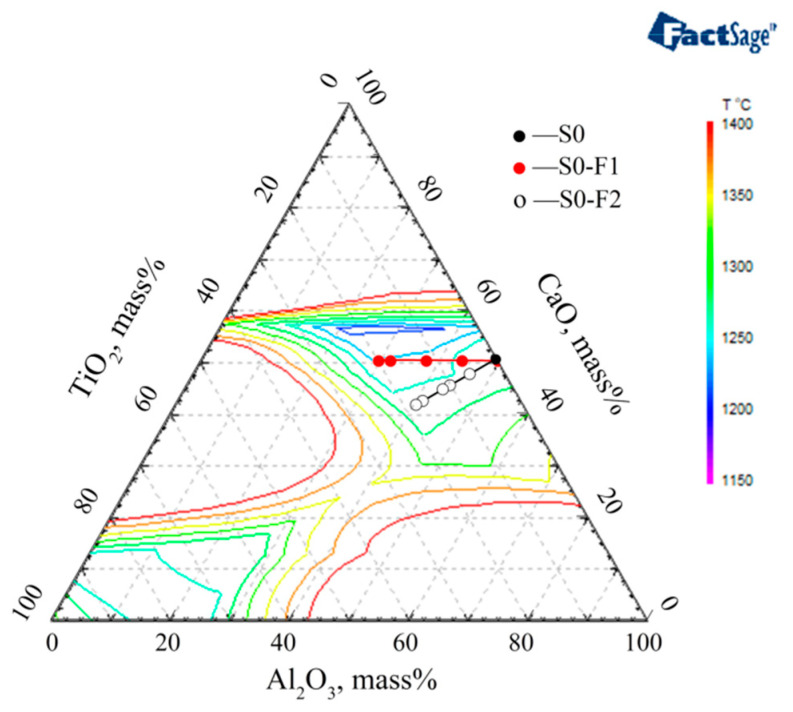
The phase diagram of CaF_2_-CaO-MgO-Al_2_O_3_-TiO_2_ (CaF_2_ = 46% and MgO = 4%).

The phenomena whereby ‘the melting temperature of the slag S0F2 system decreases first and then increases with the increase of TiO_2_ content’ and ‘the melting temperature of the slag S0F1 system decreases with the increase of TiO_2_ content’ is further explained according to the phase diagram of CaF_2_-CaO-MgO-Al_2_O_3_-TiO_2_ (CaF_2_ = 46% and MgO = 4%) calculated by Factsage, as shown in Figure 6. It can be seen that slag S0-F1 is closer to the low melting point region with the increase of F1:S0 ratio. TiO_2_ addition from 0 to 6 mass% promotes S0-F2 to approach the low melting point region, whereas the further TiO_2_ addition up to 9 mass% makes S0-F2 away from the low melting point region.

### 3.3. The Optimized Low Melting Temperature Slag Used for Steel Containing Ti and Al

**Figure 7:**
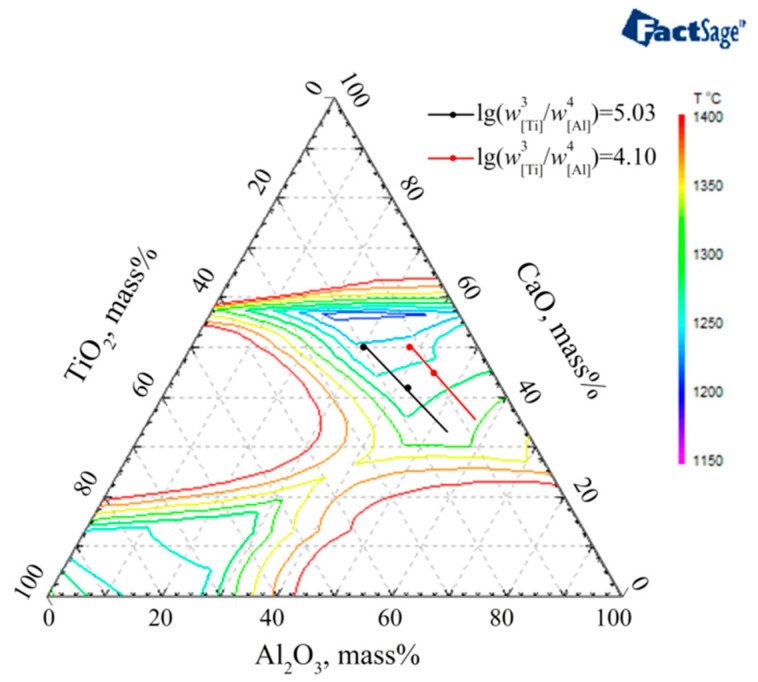
The phase diagram of melting temperature and isoactivity lines of $\mathrm{lg}\left(a_{{\mathrm{TiO}}_{2}}^{3}/a_{{\mathrm{Al}}_{2}O_{3}}^{2}\right)$ in CaF_2_-CaO-MgO-Al_2_O_3_-TiO_2_ (CaF_2_ = 46% and MgO = 4%).

It is final goals to acquire the optimized slag with low melting temperature under the condition of fixing $\mathrm{lg}\left(w_{\left[\mathrm{Ti}\right]}^{3}/w_{\left[\mathrm{Al}\right]}^{4}\right)$ ratio between Ti and Al contents in steel. During ESR of steel containing $\mathrm{lg}\left(w_{\left[\mathrm{Ti}\right]}^{3}/w_{\left[\mathrm{Al}\right]}^{4}\right)\;$= 5.03 and 4.10, the $\mathrm{lg}\left(a_{{\mathrm{TiO}}_{2}}^{3}/a_{{\mathrm{Al}}_{2}O_{3}}^{2}\right)$ in slag can be calculated as −4.51 and −5.44 according to Equations (2) and (3). Then the corresponding isoactivity lines of $\mathrm{lg}\left(a_{{\mathrm{TiO}}_{2}}^{3}/a_{{\mathrm{Al}}_{2}O_{3}}^{2}\right)\;\;$= −4.51 and −5.44 are calculated by Factsage software, as shown in Figure 7. It can be seen that slag mixtures consisting of pre-melted S0 and F1, which component is CaF_2_:CaO:MgO:Al_2_O_3_:TiO_2_ = 46:25:4:(25 − *x*):*x*, have the low melting temperature property while satisfying the $\mathrm{lg}\left(w_{\left[\mathrm{Ti}\right]}^{3}/w_{\left[\mathrm{Al}\right]}^{4}\right)$ in steel. The melting temperature of CaF_2_-CaO-MgO-Al_2_O_3_-TiO_2_ slag systems would increase with the decrease of CaO content. In addition, with the increase of CaO in slag due to the reaction of 3CaF_2_ + Al_2_O_3_ = 2AlF_3_ (g) + 3CaO during long term ESR process [27], the melting temperature of slag S0-F1 would be decreased further according to Figure 7.

## 4. Conclusions

The melting temperature of two slag systems and thermodynamic equilibrium of 3[Ti] + 2(Al_2_O_3_) = 4[Al] + 3(TiO_2_) in resistance furnace have been experimentally carried out based on phase diagram, Factsage, and thermodynamic calculation. The results are as follows:

(1) The calculated results of thermodynamic analysis based on Factsage are in good agreement with the slag-metal reaction experimental results in resistance furnace. The changes of S0:F1 and S0:F2 ratios in slag mixtures with different titanium and aluminum contents in steel are determined. The slag S0-F1 containing high CaO needs large ratio of TiO_2_/Al_2_O_3_ to guarantee the thermodynamic equilibrium of 3[Ti] + 2(Al_2_O_3_) = 4[Al] + 3(TiO_2_) and the ratio of Ti/Al in steel.
(2) The melting temperature of slag S0-F1 with a CaO/(Al_2_O_3_ + TiO_2_) ratio = 1 is lower than that of slag S0-F2 with a CaO/Al_2_O_3_ ratio = 1. Especially for thermodynamic equilibrium slag containing high TiO_2_, the melting temperature of S0-F1 slag CaF_2_:CaO:MgO:Al_2_O_3_:TiO_2_ = 46:25:4:15:10 is much lower than that of S0-F2 slag CaF_2_:CaO:MgO:Al_2_O_3_:TiO_2_ = 46:20.875:4:20.875:8.25.
(3) The slag mixtures consisting of pre-melted slag S0 (CaF_2_:MgO:CaO:Al_2_O_3_ = 46:4:25:25) and pre-melted slag F1 (CaF_2_:MgO:CaO:TiO_2_ = 46:4:25:25), which component is CaF_2_:CaO:MgO:Al_2_O_3_:TiO_2_ = 46:25:4:(25 − *x*):*x*, have the desired low melting temperature property while satisfying the concentrations of Ti and Al in steel.

## Figures and Tables

**Table 1:** Chemical composition of the SUS321 (Mass pct).

C	Si	Mn	Cr	Ni	Ti	Al	P	S
0.09	0.64	1.06	18.32	9.76	0.47	0.068	0.021	0.0017

**Table 2:** Chemical composition of slag consisting of S0 and F1(F2) used in slag-metal reaction experiments.

Exp.	Slag	CaF_2_	CaO	MgO	Al_2_O_3_	TiO_2_
S0F1-82	S0:F1 = 8:2	46	25	4	20	5
S0F2-82	S0:F2 = 8:2	46	22.5	4	22.5	5
S0F1-64	S0:F1 = 6:4	46	25	4	15	10
S0F2-64	S0:F2 = 6:4	46	20	4	20	10

**Table 3:** The results of melting temperature of each slag consisting of S0 and F1(F2).

Slag	Slag ratio	CaF_2_	CaO	Al_2_O_3_	MgO	TiO_2_	Halfsphere Temperature, K	Flowing Temperature, K
S0	--	46	25	25	4	0	1560	1570
F1	--	46	25	0	4	25	1605	1614
F2	--	46	12.5	12.5	4	25	1618	1629
S0F1-1	S0:F1 = 88:12	46	25	22	4	3	1543	1554
S0F1-2	S0:F1 = 76:24	46	25	19	4	6	1534	1546
S0F1-3	S0:F1 = 64:36	46	25	16	4	9	1533	1539
S0F1-4	S0:F1 = 60:40	46	25	15	4	10	1535	1542
S0F2-1	S0:F2 = 88:12	46	23.5	23.5	4	3	1548	1559
S0F2-2	S0:F2 = 79:21	46	22.375	22.375	4	5.25	1550	1559
S0F2-3	S0:F2 = 76:24	46	22	22	4	6	1549	1560
S0F2-4	S0:F2 = 67:33	46	20.875	20.875	4	8.25	1566	1576
S0F2-5	S0:F2 = 64:36	46	20.5	20.5	4	9	1572	1581

**Table 4:** The chemical composition of steel and slag after slag-metal reaction experiments (Mass pct).

Exp.	Si	Ti	Al	Al_2_O_3_	TiO_2_	MgO	$\mathrm{lg}\frac{f_{\mathrm{Al}}^{4}\cdot \gamma_{{\mathrm{TiO}}_{2}}^{3}}{f_{\mathrm{Ti}}^{3}\cdot \gamma_{{\mathrm{Al}}_{2}O_{3}}^{2}}$
S0F1-82	0.68	0.33	0.058	18.91	4.75	9.84	−3.57
S0F2-82	0.69	0.35	0.053	21.28	4.64	10.69	−3.21
S0F1-64	0.66	0.40	0.032	14.39	9.51	9.61	−3.42
S0F2-64	0.68	0.42	0.028	18.91	9.37	10.93	−2.89

**Table 5:** Activity interaction coefficient $e_{i}^{j}$ of the constituent in the present work.

$e_{i}^{j}$	C	Si	Mn	P	S	Al	Ti	Cr	Ni
Al	0.091	0.056	0.035	0.033	0.035	0.08	0.004	0.03	-
Ti	−0.19	−0.025	−0.043	−0.0064	−0.27	0.0037	0.013	0.055	0.009

**Table 6:** The relationship between $\mathrm{lg}\left(w_{\left[\mathrm{Ti}\right]}^{3}/w_{\left[\mathrm{Al}\right]}^{4}\right)$ and slag listed in Table 2 determined by experiments.

Slag	CaF_2_	CaO	MgO	Al_2_O_3_	TiO_2_	$\mathrm{lg}\frac{f_{\mathrm{Al}}^{4}\cdot \gamma_{{\mathrm{TiO}}_{2}}^{3}}{f_{\mathrm{Ti}}^{3}\cdot \gamma_{{\mathrm{Al}}_{2}O_{3}}^{2}}$	$\;\mathrm{lg}\left(w_{\left[\mathrm{Ti}\right]}^{3}/w_{\left[\mathrm{Al}\right]}^{4}\right)$
S0:F1 = 8:2	46	25	4	20	5	−3.33	3.76
S0:F2 = 8:2	46	22.5	4	22.5	5	−2.95	4.04
S0:F1 = 6:4	46	25	4	15	10	−3.20	5.03
S0:F2 = 6:4	46	20	4	20	10	−2.52	5.49

**Table 7:** The slag used for steel with $\mathrm{lg}\left(w_{\left[\mathrm{Ti}\right]}^{3}/w_{\left[\mathrm{Al}\right]}^{4}\right)$ = 5.03 or 4.10.

$\mathrm{lg}\left(w_{\left[\mathrm{Ti}\right]}^{3}/w_{\left[\mathrm{Al}\right]}^{4}\right)$	$\mathrm{lg}\left(a_{{\mathrm{TiO}}_{2}}^{3}/a_{{\mathrm{Al}}_{2}O_{3}}^{2}\right)$	Slag	Slag Ratio	CaF_2_	CaO	MgO	Al_2_O_3_	TiO_2_
5.03	−4.51	S0F1-4	S0:F1 = 60:40	46	25	4	15	10
5.03	−4.51	S0F2-4	S0:F2 = 67:33	46	20.875	4	20.875	8.25
4.10	−5.44	S0F1-2	S0:F1 = 76:24	46	25	4	19	6
4.10	−5.44	S0F2-2	S0:F2 = 79:21	46	22.375	4	22.375	5.25

## Data Availability

Not applicable.
